# Primary Care Interventions in Male Mental Health and Suicide Prevention: A Systematic Review

**DOI:** 10.7759/cureus.94944

**Published:** 2025-10-19

**Authors:** Anas E Ahmed, Maryam K Magfouri, Abdullah S Alamer, Noor A Alqurqush, Taif H Alzayedi, Reem A Alshehri, Fatima H Al Marar, Hissah A Alyabis, Faris A Neazy, Mohammed A Alahmari

**Affiliations:** 1 Community Medicine, Jazan University, Jazan, SAU; 2 College of Medicine, Jazan University, Jazan, SAU; 3 College of Medicine, King Faisal University, Al Ahsa, SAU; 4 College of Medicine, Taif University, Taif, SAU; 5 College of Medicine, Wrocław University, Wrocław, POL; 6 College of Medicine, King Saud bin Abdulaziz University for Health Sciences, Riyadh, SAU; 7 College of Medicine, King Saud bin Abdulaziz University for Health Sciences, Jeddah, SAU; 8 College of Medicine, King Abdulaziz University, Jeddah, SAU

**Keywords:** alcohol misuse, clinician education, general practitioners, male suicide, men's health, mental health, primary care, screening, suicide prevention, suicide risk

## Abstract

This systematic review evaluated the effectiveness of primary care interventions in improving male mental health and preventing suicide. Following the Preferred Reporting Items for Systematic Reviews and Meta-Analyses (PRISMA) guidelines, major medical databases were searched from inception to September 2025 using terms related to male populations, primary care, suicide, mental health, and substance use. After screening over 11,000 unique records and assessing 78 full texts, nine studies met the inclusion criteria. Community-based multilevel programs incorporating general practitioner training were associated with reductions in male suicide mortality compared with control regions and national trends. Tailored primary care programs, including digital tools and men's well-being initiatives, improved disclosure of suicidality, psychological distress, and overall well-being, with some demonstrating cost benefits. Interventions addressing alcohol misuse within primary care significantly reduced harmful drinking and increased abstinence, although their effects on suicidality were mixed. Overall, evidence supports the pivotal role of primary care in suicide prevention for men through clinician education, structured screening, gender-sensitive service delivery, alcohol interventions, and digital engagement tools. Sustained implementation and integration of these approaches are essential to maintain effectiveness and enhance generalizability across primary care settings.

## Introduction and background

Suicide is a major global public health concern, with men disproportionately affected. In most countries, men are at least twice as likely as women to die by suicide, despite women reporting higher rates of depression and help-seeking for mental health problems [1]. This gender paradox reflects social and behavioral factors, including traditional masculine norms, stigma, and reluctance to disclose psychological distress. Middle-aged men bear the highest burden, often linked to social isolation, unemployment, substance misuse, and limited engagement with healthcare services. Addressing these disparities requires approaches that recognize men's unique risk factors and barriers to care [2].

Primary care is a critical setting for suicide prevention and early mental health intervention. General practitioners (GPs) are often the first point of contact for individuals in distress, and many men who die by suicide have consulted a GP in the months before their death. This creates a key opportunity for risk detection and timely intervention [3]. Primary care also provides continuity of care and access for men less likely to engage with specialist mental health services. However, under-recognition of suicide risk, time pressures, and men's reluctance to discuss mental health remain significant challenges [1].

A range of interventions has been developed to improve men's mental health and reduce suicide risk in primary care. These include GP training programs to enhance the detection and management of depression, community-based strategies integrating primary care with public awareness campaigns, and consultation tools designed to support men's disclosure of distress [4]. Other promising approaches include brief counselling for alcohol misuse, male-sensitive well-being services, and digital activation programs aimed at improving engagement and help-seeking. Evaluating these interventions is essential to identify effective strategies for suicide prevention among men [5].

Despite growing research, the evidence remains fragmented. Studies vary widely in design, quality, and outcomes, with mixed findings regarding reductions in suicide or improved engagement among high-risk men. Few reviews have focused specifically on interventions targeting men within primary care, despite its central role in prevention. Synthesizing this evidence is crucial to guide clinicians, policymakers, and service planners in implementing effective, scalable approaches [2,6,7].

This systematic review, therefore, aims to evaluate and synthesize evidence on interventions delivered in or linked to primary care that address men's mental health and suicide prevention. By focusing on male populations and primary care contexts, the review seeks to identify strategies that enhance early detection, treatment engagement, and risk reduction while informing clinical practice, policy, and future research priorities.

## Review

Methodology

Literature Search Strategy

This systematic review followed the Preferred Reporting Items for Systematic Reviews and Meta-Analyses (PRISMA) guidelines [8]. A comprehensive search was conducted in PubMed, Cochrane Library, Scopus, and Web of Science from database inception to September 2025. The search combined controlled vocabulary and free-text terms related to men and masculinity, primary care and general practice, suicide and suicidal behavior, mental health (e.g., depression, anxiety), and associated risk factors such as alcohol and substance misuse. Search terms were adapted for each database using Boolean operators. Filters were applied to include only English-language studies involving human male participants. Reference lists of included articles were also manually screened to identify additional eligible studies.

Eligibility Criteria

Eligibility was defined using the PICOS (Population, Intervention, Comparator, Outcome, Study design) framework [9]. Studies were included if they involved male participants or reported male-specific data; were conducted in primary care or community settings linked to general practice; evaluated interventions targeting mental health, suicide prevention, or related risk factors such as alcohol misuse; reported male-specific outcomes, including suicide, suicidal ideation, mental health symptoms, well-being, or help-seeking; and used randomized controlled, quasi-experimental, cohort, case-control, or service evaluation designs. Exclusion criteria were studies unrelated to men or primary care, descriptive epidemiology without intervention, non-English publications, conference abstracts, and reviews or editorials.

Study Selection

Two reviewers independently screened all titles and abstracts against the inclusion criteria. Full texts of potentially eligible articles were reviewed to confirm inclusion. Discrepancies were resolved through discussion, and a third reviewer was consulted when necessary.

Data Extraction

Data were extracted independently by two reviewers using a standardized form. Extracted variables included study identification (author, year), country and setting, design, participant characteristics, intervention type, comparator, male-specific outcomes, and main findings. Missing data were sought from supplementary materials when available.

Quality Appraisal

The methodological quality of included studies was assessed using the Mixed Methods Appraisal Tool (MMAT) [10], applicable to randomized, non-randomized, observational, and mixed-methods designs. Each study was evaluated using five design-specific criteria in addition to screening questions. Studies were classified as high quality if they met most or all criteria with minimal risk of bias or moderate quality when concerns existed regarding confounding, representativeness, or rigor. Two reviewers conducted appraisals independently, resolving disagreements through discussion until consensus was achieved.

Results

Study Selection

**Figure 1:**
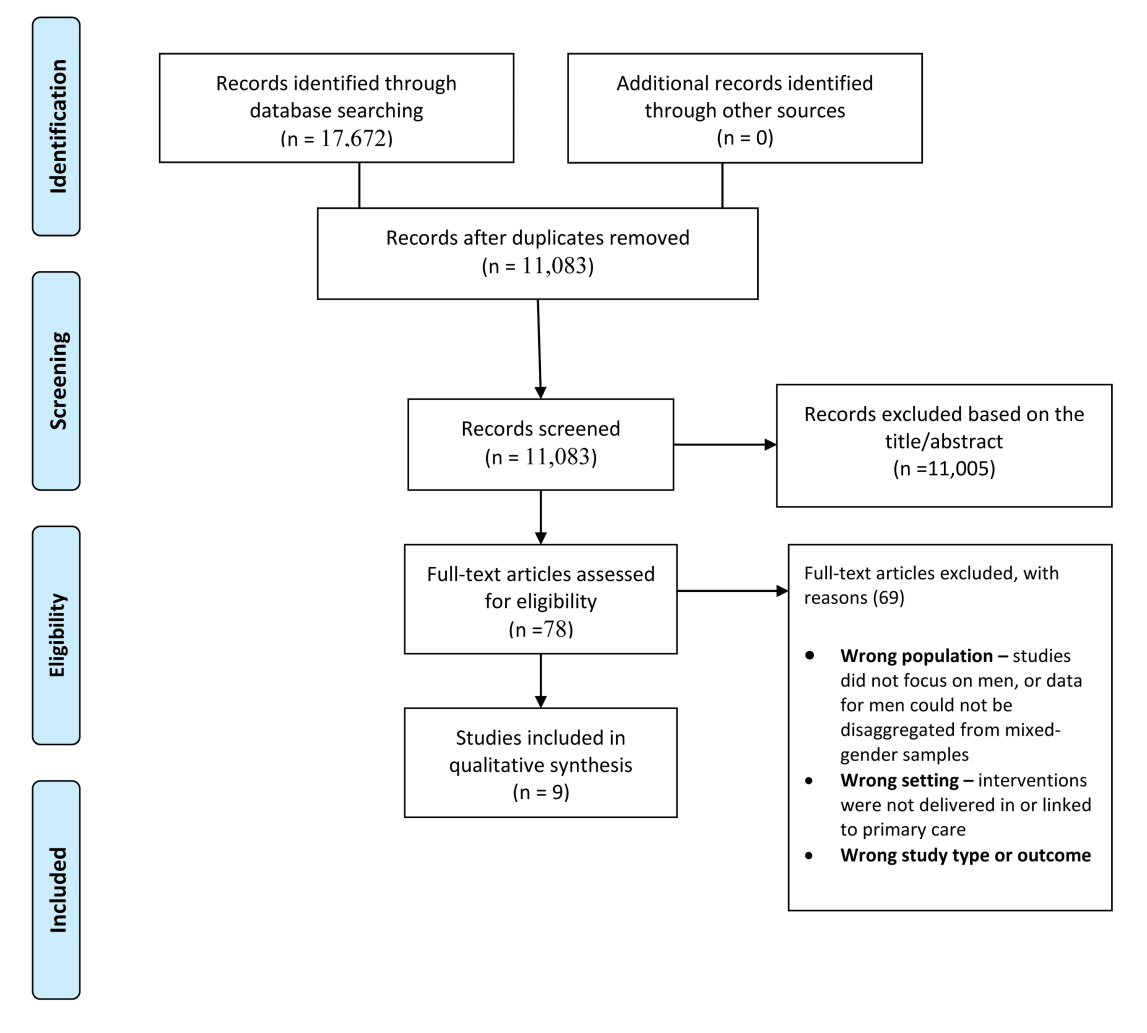
PRISMA flow diagram showing the study selection PRISMA: Preferred Reporting Items for Systematic Reviews and Meta-Analyses

A total of 17,672 records were identified through database searches (PubMed=3,549; Cochrane=3,911; Scopus=5,678; Web of Science=4,534), with no additional records retrieved from manual or grey literature searches. After removing duplicates, 11,083 unique records were screened, of which 11,005 were excluded based on titles and abstracts. Seventy-eight full texts were assessed, and 69 were excluded for reasons including non-male-focused populations, settings unrelated to primary care, or inappropriate study design or outcomes. Nine studies met the inclusion criteria and were included in the qualitative synthesis [1-7,11,12] (Figure 1).

Study Characteristics

The included studies evaluated a range of primary care and community-based interventions addressing male mental health and suicide prevention. Population-level initiatives in Hungary integrated GP training, specialist support, and public awareness to reduce suicide mortality, though alcohol-related deaths among men persisted [3,7]. In Switzerland, a primary care-linked campaign tailored for gay men improved mental health literacy, distress, and suicidality [5]. These programs highlight the potential of GP-centered and community-linked models to enhance male help-seeking and mental health outcomes (Table 1).

**Table 1:** Summary of the included studies evaluating primary care-based interventions and service models for male suicide prevention GP: general practitioner; PHC: primary health center; RCT: randomized controlled trial; PCC: primary care clinic; SES: socioeconomic status; IQR: interquartile range; BME: Black and minority ethnic; AUDIT: Alcohol Use Disorders Identification Test; EAAD: European Alliance Against Depression; MAPS: Men and Providers Preventing Suicide; CAP: Counselling for Alcohol Problems; CBT: cognitive behavioral therapy; EUC: Enhanced Usual Care; WHO: World Health Organization; mhGAP: Mental Health Gap Action Programme; CIDI-SF: Composite International Diagnostic Interview-Short Form; MHI-5: Mental Health Inventory-5; K6: Kessler Psychological Distress Scale; HADS: Hospital Anxiety and Depression Scale; PSS: Perceived Stress Scale; WEMWBS: Warwick-Edinburgh Mental Well-being Scale; PSYCHLOPS: Psychological Outcome Profiles; PHQ-9: Patient Health Questionnaire-9; IPV: intimate partner violence; SSRIs/SNRIs: selective serotonin reuptake inhibitors/serotonin-norepinephrine reuptake inhibitors; NS: not significant; OR: odds ratio; aOR: adjusted odds ratio; aPR: adjusted prevalence ratio

Study ID (author)	Country/setting	Study design	Male participants (N, age, demographics)	Intervention type	Comparator	Male-specific outcomes	Male-specific results
Mughal et al. [1]	UK (England, Scotland, Wales)/primary care+coroners' data	National consecutive case series (descriptive epidemiology, 2017 deaths)	1,516 male suicides aged 40-54 years (¼ of all suicides that year). Sample N=288 (20%); data for N=242 (mean age 47). The majority are unemployed/low-income; half live alone	Not a trialled intervention; examined GP consultations in 3 months before suicide as a window for primary care intervention	Males with GP contact ≤3 months vs. >3 months (or none) before suicide	Timing of last GP consultation; presenting problems (mental health, physical illness, work-related); prescriptions; antecedents (self-harm, suicidal ideation, stressors)	43% had a GP consultation within 3 months before suicide; 12% within 1 week. Of these, 51% presented with a mental health problem. Predictors of recent GP contact: self-harm (OR=3.49; p=0.002), major illness (OR=2.39; p=0.004), mental health problem (OR=4.92; p<0.001), and work stress (OR=3.07; p=0.004). Men with recent GP contact are more likely to be prescribed SSRIs/SNRIs (53% vs. 23%), antipsychotics (19% vs. 8%), and benzodiazepines (14% vs. 5%). Highlights primary care as critical for suicide prevention in middle-aged men
Stanistreet et al. [2]	UK/Merseyside and Cheshire (coroner and GP data)	Retrospective epidemiological study	268 men aged 15-39 years with sudden violent deaths (97 suicides/undetermined, 130 accidental, 41 excluded)	Analysis of GP/hospital contacts before death (proxy for preventive opportunities in primary care)	Comparison of accidental vs. suicide/undetermined deaths	GP contact in the last 1-3 months; prior mental health service contact; content of GP visit	56% of suicide cases vs. 41% of accidental deaths saw a GP within 3 months (p=0.05). 38% of suicides vs. 30% accidents saw a GP within 1 month (NS). 27% of suicide cases had ever seen a mental health worker (vs. 13% of accidents). Many consultations related to substance misuse; suicides are more often linked to psychological problems. Demonstrates low GP engagement among young men before suicide
Székely et al. [3]	Hungary/Szolnok (community-based)	Quasi-experimental, community intervention	Male population: 36,314 men (of 76,881 total). Age not specified	EAAD four-level intervention: (1) GP training; (2) public awareness campaign; (3) community facilitator education; (4) hotline and self-help support	Control region (Szeged)+national suicide rates	Male suicide rate per 100,000; help-seeking behaviors	Male suicide rates decreased from ~45.5/100k (2002-2004) to 18/100k (2005-2007) → ~60% reduction. Reduction is similar in men and women, unlike many other programs. ↑ Hotline calls (many anonymous, appealing to men). ↑ Psychiatric outpatient visits
Jerant et al. [4]	USA/California (8 primary care offices, university-affiliated health system)	Cluster RCT (PCC-level randomization)	N=48 men (35-74 years) with recent (≤4 weeks) suicidal thoughts; mean age ~56; majority White, most married, mixed income levels	MAPS: tailored multimedia computer program (15-20 min pre-visit) designed to overcome male-specific barriers (stigma, toughness, fear of hospitalization)	Active control: 3-minute sleep hygiene video+generic text encouraging suicide discussion	Primary: whether suicide was discussed during the subsequent GP visit. Exploratory: effect moderation by preparatory behaviors	Suicide discussion is more likely in MAPS (65%) vs. control (35%). OR 5.91 (95% CI 1.59-21.94; p=0.008); adjusted predicted effect 71% vs. 30%. Among men with preparatory behaviors: 92% MAPS vs. 33% control discussed suicide (OR 27.45; p=0.005). No effect in men without preparatory behaviors. Conclusion: MAPS effectively activated men to disclose suicidal thoughts in primary care
Wang et al. [5]	Switzerland/Geneva (urban, community-based)	Pre- and post-intervention surveys (2007 vs. 2011) of gay men	2007: N=276 gay men (mean age mid-30s; more educated, urbanized, single vs. general male population). 2011: N=486 gay men	Blues-out Campaign (adapted EAAD model): cooperation with primary care physicians, depression awareness campaign (posters, brochures, website), hotline/emergency cards, gay-specific materials	Pre-intervention (2007) vs. post-intervention (2011); subgroup comparisons of men aware vs. unaware of the campaign	Mental health literacy (recognition of depression, attitudes, help-seeking beliefs); suicidality (ideation, plans, attempts); depression and distress (CIDI-SF, MHI-5, K6)	No change in overall recognition of depression (43% → 43%). Significant decreases in suicidality: lifetime suicidal ideation ↓ from 55.8% to 47.8% (p=0.04); suicide plans ↓ from 38.5% to 27.5% (p=0.003); attempts stable (~17%). Lifetime chronic depression ↓ 57% → 47% (p=0.01). High psychological distress ↓ 29.2% → 21% (p=0.015). Men aware of the campaign are more likely to recognize depression (55% vs. 42%) and value psychiatric help. No control group; small effect sizes
Nadkarni et al. [6]	India/10 PHCs (Goa)	RCT	N=377 harmful male drinkers (18-65 years; mean ~42; mostly married, manual laborers, low SES)	CAP: brief manualized intervention (motivational interviewing, behavioral and cognitive techniques), delivered by trained lay counsellors in PHCs (≤4 sessions)	EUC: GP consultation+WHO mhGAP harmful drinking guidelines	AUDIT remission <8; daily alcohol use; abstinence; suicide attempts	Higher remission (36% vs. 26%; p=0.01); more abstinent (42% vs. 18%; p<0.0001); increased days abstinent (p<0.0001); no effect on suicide attempts (none vs. 3; NS). Cost-effective ($217/additional remission)
Szanto et al. [7]	Hungary/Kiskunhalas (rural+town)	Quasi-experimental, GP-based intervention	Intervention region: 73,000 (majority rural). Male data emphasized: high baseline suicide rates (~60/100k)	5-year GP-based depression management program: GP and nurse training, depression clinic, psychiatrist phone consultations, expedited antidepressant access	Local control region, surrounding county, and national rates	Male suicide rate, antidepressant use, and alcohol-related deaths	Male suicide rates declined significantly (greater reduction than county/national rates; p<0.001). Rural men had particularly high baseline rates, but the decline was stronger in women. Alcoholism is prevalent in 75% of male suicides; limited effect on alcohol-related deaths. Highlights the need for male-specific approaches addressing alcohol+depression
Cheshire et al. [11]	UK/London (Victoria Medical Centre, large GP practice)	Pilot service evaluation (pre-post, no control)	N=102 men, median age 41 (IQR 31-49), broad ethnic mix (48% White British, 24% BME), 18% unemployed, ⅔ using medication/alcohol to cope with stress	Atlas Men's Well-being Programme: GP referral of distressed men → up to 12 counselling and/or 6 acupuncture sessions. Male-sensitive service (male-only, flexible options, supportive language)	No comparator (pre-post only)	Anxiety (HADS); depression (HADS); stress (PSS); well-being (WEMWBS); patient-generated outcomes (PSYCHLOPS); physical health; costs (employment loss, healthcare use)	Significant improvement in anxiety (p<0.001), stress (p<0.001), well-being (p<0.001), PSYCHLOPS (p<0.001), and physical health (p=0.001). No overall change in depression (p=0.660), but significant improvement among the depressed subgroup (p<0.001). Counselling is more effective than acupuncture; combined not superior. 78% reported feeling better post-treatment. Cost savings ≈ £700/patient. GPs pivotal in identifying and referring men
Nadkarni et al. [12]	India/Goa-10 PHCs	RCT (12-month follow-up)	N=377 harmful male drinkers (AUDIT 12-19), aged 18-65; mostly low SES, manual laborers, majority married. Mean age ~42	CAP: brief manualized psychological intervention (motivational interviewing, CBT, relapse prevention) by trained lay counsellors (≤4 sessions)	EUC: GP consultation+WHO mhGAP guidelines for harmful drinking	Primary: remission (AUDIT <8), mean ethanol consumption (14 days). Secondary: abstinence, recovery, % days abstinent/heavy drinking, PHQ-9 depression, suicidal behavior, disability, IPV, costs	Remission at 12 months higher with CAP+EUC (54.3%) vs. EUC (31.9%) (aPR 1.71; p<0.001). Abstinence higher (45.1% vs. 26.4%; aOR 1.92; p=0.008). Recovery (27.4% vs. 15.1%; aPR 1.90; p=0.006). % days abstinent higher (71% vs. 55%; p=0.001). No significant effect on PHQ-9, suicidal behavior (8.6% vs. 11.2%), or IPV. Cost-effective: CAP dominant, cost-saving (72-94% analyses). Readiness to change at 3 months predicted reduced drinking at 12 months

Observational studies from the United Kingdom demonstrated that many men who died by suicide had recent GP contact, reflecting missed opportunities for prevention. Coroner-linked analyses found that nearly half of middle-aged men who died by suicide had visited a GP within three months, often for psychological, physical, or work-related concerns [1,2]. These findings emphasize the need for systematic suicide risk assessment and structured follow-up in primary care.

Targeted, male-sensitive interventions showed promising effects. In the United States, a cluster randomized controlled trial (RCT) found that a pre-visit digital tool increased suicide-related discussions between men and physicians [4]. In India, two RCTs of brief alcohol counselling delivered by lay counsellors in primary care improved remission and abstinence rates and demonstrated cost-effectiveness [6,12]. In the United Kingdom, a male-sensitive well-being service improved anxiety, stress, and well-being, with evidence of cost savings [11].

Quality Assessment

Of the nine studies, three RCTs were rated as high quality. The Indian RCTs evaluating the Counselling for Alcohol Problems intervention demonstrated strong methodological rigor with appropriate randomization, complete data, and fidelity monitoring [6,12]. Similarly, the cluster RCT assessing the digital activation tool used a robust design with balanced groups and low attrition [4] (Table 2).

**Table 2:** Quality appraisal of the included studies evaluating primary care and community interventions for male suicide prevention The quality appraisal was done using the Mixed Methods Appraisal Tool (MMAT) [10]. GP: general practitioner; RCT: randomized controlled trial; MAPS: Men and Providers Preventing Suicide; PCC: primary care clinic; CAP: Counselling for Alcohol Problems; PHC: primary health center

Study ID (author)	Design	S1: clear Qs	S2: data adequate	Representativeness/randomization	Measurements appropriate	Outcome data complete	Confounders/group comparability	Intervention delivered as intended/blinding	Overall appraisal
Mughal et al. [1]	Observational case series (GP consultations pre-suicide)	Yes	Yes	Yes	Yes	Partial	Partial	Yes	Moderate
Stanistreet et al. [2]	Retrospective epidemiological (observational)	Yes	Yes	Yes	Partial	Partial	No	Yes	Moderate
Székely et al. [3]	Quasi-experimental (community, non-randomized)	Yes	Yes	Yes	Yes	Yes	Partial	Yes	Moderate
Jerant et al. [4]	RCT (MAPS tailored activation, US PCCs)	Yes	Yes	Yes (cluster randomization)	Yes	Yes	Yes	Yes	High
Wang et al. [5]	Pre-post survey (no control)	Yes	Yes	Yes	Partial	Partial	No	Partial	Moderate
Nadkarni et al. [6]	RCT (CAP intervention, PHCs, Goa)	Yes	Yes	Yes (randomization appropriate)	Yes	Yes	Yes	Yes	High
Szanto et al. [7]	Quasi-experimental (GP education and depression clinic)	Yes	Yes	Yes	Yes	Yes	Partial	Partial	Moderate
Cheshire et al. [11]	Pilot service evaluation (GP-referral well-being program)	Yes	Yes	Yes	Partial	Partial	No	Yes	Moderate
Nadkarni et al. [12]	RCT (CAP intervention, PHCs, Goa; 12-month follow-up)	Yes	Yes	Yes (randomization concealed)	Yes	Yes	Yes	Yes	High

The six non-randomized or observational studies were rated as moderate quality. Quasi-experimental Hungarian programs provided strong population-level data but limited adjustment for confounders [3,7]. Observational UK studies had issues with representativeness and incomplete data [1,2,11]. The pre-post survey from Switzerland lacked a control group and had low response rates, limiting inference [5].

Effect of Interventions on Suicide Mortality (Population-Level Programs)

Community-based multilevel programs with strong primary care components were consistently linked to lower suicide rates. In Hungary, a four-level model incorporating GP education and referral pathways led to significant reductions in male suicides compared with control regions and national trends, though effects diminished over time [3,7]. These programs also increased antidepressant prescribing and psychiatric referrals, but had a limited impact on alcohol-related deaths.

Effect of Interventions on Suicidality, Disclosure, and Help-Seeking

UK data showed that 40-45% of men who died by suicide had recent GP contact, often presenting with self-harm, psychological distress, or occupational stressors [1,2]. In a cluster RCT, a brief digital pre-visit tool more than doubled the likelihood of suicide-related discussion between men and their physicians, especially among those with preparatory behaviors [4].

Effect of Interventions on Mental Health Symptoms and Well-Being

A GP-referral male well-being program offering counselling and acupuncture improved anxiety, stress, and well-being and appeared cost-saving through reduced healthcare use and productivity losses, though effects on depression were limited [11]. A campaign targeting gay men and integrated with primary care reduced suicidal ideation and distress while improving mental health literacy, though causal inference was limited by the absence of a control group [5].

Effect of Interventions on Alcohol Use Interventions

Two RCTs in Indian primary care demonstrated that brief counselling for harmful drinking significantly increased remission and abstinence and sustained benefits at 12 months [6,12]. Although effects on depression and suicidality were neutral, these programs addressed a key modifiable suicide risk factor among men.

Effect of Interventions on Service Utilization and System Effects

Programs that trained GPs and strengthened community linkages increased psychiatric outpatient visits and hotline use, particularly among men, suggesting improved help-seeking pathways [3]. Observational UK findings similarly indicated that men with recent GP contact before suicide had higher referral and emergency attendance rates, highlighting actionable clinical signals for proactive intervention [1].

Discussion

This review found that primary care-linked interventions can reduce male suicide risk through multiple pathways. Multicomponent community programs that embedded GP education and referral mechanisms were associated with population-level declines in suicide mortality [13,14]. At the consultation level, men who later died by suicide often had recent GP contact, revealing a window for targeted assessment and follow-up. Male-tailored clinical tools, such as brief pre-visit digital activation, increased disclosure of suicidality during primary care visits, while male-sensitive services improved anxiety, stress, and well-being and appeared cost-saving. Brief alcohol interventions delivered in primary care produced robust improvements in harmful drinking and were often cost-effective, addressing a major modifiable risk factor for male suicide [13-15].

Our findings align with universal standards for suicide prevention in primary care, which emphasize sustained clinician education, systematic screening, safety planning, lethal means counselling, and caring contacts around high-risk transitions [4,5]. Programs that trained GPs and formalized referral pathways were associated with lower mortality, and ongoing, multiyear initiatives outperformed short, standalone campaigns. GP-linked programs increased psychiatric visits and hotline use, supporting the value of embedding protocols and follow-up within primary care workflows.

Middle-aged men frequently present with self-harm, work stressors, comorbid physical illness, and psychotropic prescribing, highlighting high-risk groups. Activation tools and safety planning are plausible mechanisms for increasing disclosure and intervention in routine care. Recognition gaps in general practice underscore the importance of normalizing discussions about suicide, where male-tailored programs demonstrated benefit [4,13-15].

Multimodal programs combining awareness campaigns, gatekeeper training, and facilitated access to support appear most effective for reducing male suicide, while campaigns alone yield smaller or short-lived effects. Primary care interventions that incorporate GP education and collaboration more closely resemble effective multimodal configurations. Addressing alcohol misuse in primary care is a high-yield strategy, with brief interventions improving remission, abstinence, and recovery while being cost-effective [4,5,14,15].

Male-sensitive delivery models also enhance engagement. Programs that offer flexible, pragmatic, and respectful support, such as counselling, acupuncture, or pre-visit digital prompts, can improve well-being and encourage disclosure [14]. Gender-tailored approaches that normalize help-seeking and reduce stigma can unlock the preventive potential of routine GP visits [5,13].

Implications for practice include adopting sustained GP education, universal depression screening with targeted suicide screening for higher-risk men, codified safety planning and means reduction, caring contacts around transitions, embedded alcohol interventions, and male-tailored activation tools. At the population level, multimodal programs integrating GP training with community awareness and facilitated access appear most promising, though continuity and adequate resourcing are critical to maintain effects over time.

Limitations

Limitations of this review include heterogeneity in interventions, settings, and outcome measures, which precluded meta-analysis; reliance on several non-randomized or observational designs (e.g., community programs, case series) that may introduce confounding; limited male-specific subgroup analyses within mixed samples; and inconsistent reporting of implementation fidelity and participant exposure. Publication and language restrictions (English-language focus) may have led to the omission of relevant studies. Finally, most included interventions were conducted within specific health systems or locales, potentially limiting generalizability without contextual adaptation.

## Conclusions

Primary care serves as a pivotal platform for male suicide prevention. A comprehensive approach that combines clinician support, structured screening, proactive follow-up, and gender-sensitive service delivery offers a practical framework for reducing risk and improving well-being among men. Integrating these components into cohesive, well-resourced care pathways and maintaining them over time can help embed prevention within routine practice. Future research should focus on implementing and evaluating bundled, gender-responsive models across diverse primary care settings, with emphasis on long-term sustainability, equity, and male-relevant outcomes such as suicidality, functioning, and engagement.
